# The Fever Hospitals of the Metropolitan Asylums Board

**Published:** 1911-12-16

**Authors:** R. A. Birdwood

**Affiliations:** Medical Superintendent of the Park Hospital.


### THE FEVER HOSPITALS OF THE METROPOLITAN ASYLUMS BOARD.

By R. A. BIRDWOOD, M.A., M.D., M.R.C.S., Medical Superintendent of the Park Hospital.

Fever hospitals serve a dual purpose. The removal of the infectious sick from contact with the healthy tends to control the spread of the disease amongst the people; their admittance to a hospital supplies the sufferers with the accommodation and care not always available at home. The preventive value of isolation was recognised early in the history of the Hebrew race. Our own forefathers also adopted segregation for diseases, in their day, thought to be communicable. In both instances the care of the individual seemed to be subordinate to that of the protection of the community. No such consideration, however, influenced Parliament when the Metropolitan Asylums Board was established for making provision for the care of the infectious sick. Indeed, it is doubtful whether anyone in 1867 contemplated the possibility of a London without small-pox or the prevention by isolation of fevers generally.

### Aim of the Metropolitan Asylums Board.

To provide " asylum " for the destitute " infected with or suffering from fever, or the disease of small-pox, or maybe insane," was the primary function for which the Board was established. Admission was to be by order of the relieving officer or master of the workhouse, and the parochial medical officer had to sign the certificate. Power was given to the medical superintendent to admit any person who presented himself at the asylum without the order and certificate if he were satisfied that refusal to admit might be attended with dangerous results.

As the years passed on, the other local authorities entrusted with the duty of providing for the well-to-do infectious sick failed to make adequate provision for their hospital accommodation. The good repute of the hospitals first built by the managers at Hampstead, Stockwell, and Homerton spread rapidly, and the consequence was that they were used by others than the destitute. Fewer and fewer patients were sent in on the relieving officer's orders, and more and more presented themselves at the hospital gate to be admitted by the medical superintendent.

So it came about ultimately that destitution was no longer the passport; any qualified medical practitioner's certificate that the patient had one of the isolable diseases made that person eligible for admission if there was a bed vacant in the hospital.

### The Place of Isolation Hospitals.

The isolable diseases are mainly small-pox, diphtheria, scarlet and enteric fevers, with some others in small numbers. Recently measles and whooping-cough in poor children have been added to the list. As yet, all measles and whooping-cough patients are not eligible, but only those sent in by Poor Law officials or those recommended by Medical Officers of Health.

In addition, provision has been made, on occasion, for cholera, plague, cerebro-spinal meningitis. No payment is permitted on behalf of any patient.

We come now to this point: The community recognise that the isolation of an individual suffering from communicable disease is carried out in the interest of the many, and to secure such isolation the patient must be well cared for. The idea of asylum for the relief of the unfortunate gives way to that of a guest-house for hospital treatment during illness and entertainment till risk of communicating infection is past. The public is the host, and in London the whole and sole cost of maintenance is paid by them: this is not only right but prudent, for the money is really a sort of insurance premium against the enormous expense of a widespread epidemic.

The more thoroughly isolation is carried out, the sooner will the expenditure reach the vanishing point. If the father of a poor family has a mild attack of an infectious fever, he may conceal it and so spread the disease. If he has to pay for his maintenance during his isolation, his temptation to act thus is great, especially if he has heard of his neighbour's home having been broken up during such an enforced absence. On the other hand, the knowledge that, whilst he is in hospital there will be no charge on his purse for that stay, is an inducement to him to accept the proffered hospitality.

### An Essential of Sanitary Legislation.

Not only is no payment required, but practically (with a few exceptions) there is no compulsory removal to hospital. It is essential for the success of sanitary legislation that the co-operation of the public should be secured, and if it can be secured without compulsion so much the better. It is this absence of compulsion (for no man is required to have his sick removed if he has adequate accommodation at home) which disarmed public opposition. All classes have the right to use these hospitals; hardly any are compelled to enter, and no one may pay for their use.

These are the conditions for the success of isolation as a means of prevention in densely crowded urban communities. With them the co-operation of all can be secured. The Board's annual reports show how this co-operation has steadily improved. In the past twenty years the ratio of admissions to notifications of each admissible disease has progressively risen. It rose from about forty per cent. to ninety in scarlet fever; from twenty to eighty-five in diphtheria; and similarly with enteric and small-pox.

### Almost Complete Isolation.

The increasing popularity of the Board's hospitals has resulted in nearly complete isolation. Only within the last few years has isolation as a means of prevention had any chance; for little result could be expected if fewer of the infected were admitted than were left outside to spread the disease. It is to be hoped that soon all will consent to hospital isolation. The point to be grasped is that the purely palliative care of the sick has been abandoned as the motive of hospital isolation. The aim is not that the poor are to have a bed, but that the time may soon come when the bed will not be required by anyone having a preventable disorder. There are indications that this is coming about. For instance, the beds provided for small-pox are unoccupied for many consecutive months. Again, a previous attack of the diseases not admissible, such as chicken-pox and, till recently, measles and whooping cough, are much greater in our fever hospital population than are the admissible diseases. Out of one thousand scarlet fever patients 712 were stated to have previously had measles, 353 whooping-cough, 330 chicken-pox, and only 34 diphtheria. Out of 500 diphtheria patients, 316 had had measles, 183 whooping-cough, 159 chicken-pox, and only 44 scarlet fever. These figures indicate that the isolation of scarlet fever and diphtheria patients is preventing the incidence of these diseases.

### Area and Population under the Board.

The area of the district served by the Metropolitan Asylums Board is 121 square miles. The estimated population is approaching five millions. Ten hospitals, containing 6,110 beds, are provided for fevers; that is about one bed for every 797 persons resident in the district. Eight of the hospitals, with 3,840 beds, are in London, for acutely ill patients on removal from their homes. The other two, with 2,270 beds, are in the country; these are for scarlet fever and diphtheria convalescents from the acute hospitals.

The following table shows the geographical distribution, the accommodation, acreage of the site, and the date of opening of each of the fever hospitals. It will be noticed that the London hospitals are on the periphery of an oval following the boundary of the county, and that every house in the Metropolis is within easy reach of some one hospital:—

| Date of opening | Name | Situation | Acreage | Beds |
|---|---|---|---|---|
| 1870 | North Western ... | Hampstead | 12 | 474 |
| 1871 | South Western ... | Stockwell | 8 | 339 |
| 1871 | Eastern ... ... | Homerton | 9 | 368 |
| 1877 | Western ... .. | Fulham | 13 | 452 |
| 1877 | South Eastern ... | Deptford | 10 | 498 |
| 1892 | North Eastern ... | Tottenham | 33 | 623 |
| 1896 | Brook ... ... ... | Woolwich | 29 | 568 |
| 1899 | Grove ... ... ... | Tooting | 22 | 518 |
| | | | 136 | 3,840 |
| 1887 | Northern ... ... | Winchmore Hill | 35 | 738 |
| 1890 | Southern ... ... | Dartford | 160 | 1,532 |
| | | | 331 | 2,270 |
| | | | | 6,110 |

There are about 28 beds to an acre in the London hospitals and 18 beds to an acre if the two in the country are included.

### How Specialisation is Secured.

The extensive area of the hospital sites enables different infections to be dealt with in each hospital. The arrangement in this hospital (it was formerly used for isolation) is in widely separated pavilions of ground and first floor wards, with outside stairs, opening on to an open corridor or covered way. The groups of wards for each infection are separated by the administrative block. The wards are lighted by large windows opening up to within a couple of inches from the ceiling; for night, electric light is provided.

The heating and ventilation in cold weather are effected by inlets for radiator-warmed fresh air at alternate windows, and cold fresh air admitted through perforated bricks under each bed. The exit for used air is near the ceiling in the stack of pipes connected with the two central fireplaces in each ward.

Steam generated in the boiler-house is conveyed in a subway under the corridor to each ward, and there it warms the water in two heaters—one for the domestic supply, baths, etc., the other for the radiators. The advantage of this system is that it has been easy to maintain a constant temperature in the wards during the coldest weather of the past seventeen years. Stuffiness can also be readily avoided. The wards are 26 feet wide, and it is calculated that about 1,760 cubic feet of air space are allowed for the former and 2,000 for the latter. There are twenty beds in the scarlet fever wards and twelve in the others. To each of these wards are attached a one-bed ward and a two-bed ward for observation purposes, in the event of the diagnosis being uncertain or the prospect of a patient sickening for some other complaint. These small wards are also well suited for the treatment of members of the staff.

### Small Wards in Isolation Hospitals.

Besides these observation wards, it is considered necessary to set apart certain groups of small wards for the isolation of patients affected with more than one infectious disease; for example, a scarlet-fever patient might be incubating chicken-pox or measles at the time of admission, the eruption not appearing for some days later.

Intimation of exposure to the infection of the second disease a few days before admission being given, such patient would not be sent into one of the large wards, but would be placed in a single-bed ward on the isolation corridor. It is quite common for a child to have two infectious diseases at the same time, or such conditions as post-scarlatinal diphtheria. Again, a mistaken diagnosis or a doubtful one should not entail the patient's exposure to infection in the ward. These and other similar cases have these small wards provided for their security. At this hospital six pavilions of ten beds each, arranged in wards of four beds and one bed upstairs and the same downstairs, have given us one bed for isolation within the hospital for every eight beds for the ordinary patients; or if we include the 72 beds for observation purposes, we have a little less than one-quarter of the hospital accommo-[illegible]on set apart for doubts, mistakes, and co-existing infections. This large number of beds is necessary as shown by the fact that all of them are sometimes in occupation.

*(To be continued.)*

